# Synthesis and Analgesic Activity of Novel Hydrazide and Hydrazine Derivatives

**Published:** 2013

**Authors:** Mansur Nassiri Koopaei, Mohammad Javad Assarzadeh, Ali Almasirad, Seyedeh Farnaz Ghasemi-Niri, Mohsen Amini, Abbas Kebriaeezadeh, Nasser Nassiri Koopaei, Maryam Ghadimi, Arash Tabei

**Affiliations:** a *Department of Medicinal Chemistry, Pharmaceutical Sciences branch, Islamic Azad University, Tehran, Iran. *; b *Department of Pharmacology and Toxicology, Faculty of Pharmacy and Pharmaceutical Sciences Research Center, Tehran University of Medical Sciences, Tehran, Iran. *; c *Department of Medicinal Chemistry, Faculty of Pharmacy and Pharmaceutical Sciences Research Center, Tehran University of Medical Sciences, Tehran, Iran. *

**Keywords:** Hydrazide, Hydrazone, Analgesic activity, Fenamate

## Abstract

The uses of non-steroidal anti-inflammatory drugs (NSAIDs) are limited by a variety of side effects. So research on preparing new analgesic agents is important. According to some reports about the analgesic activity of hydrazide and hydrazine derivatives a new series of these compounds were synthesized in order to obtain new analgesic compounds. The final compounds 10a-10e and 15a-15d were prepared by condensation of corresponding hydrazides 7,8 and 11-14 with different aldehydes 9a-9e. The structures of all synthesized compounds were confirmed by means of FT-IR, 1H-NMR and Mass spectra. All compounds were evaluated for their analgesic activities by abdominal constriction test (writhing test). Most of the synthesized compounds induced significant reduction in the writhing response when compared to control and compound 15 was more potent than mefenamic acid in the writhing test.

## Introduction

Non-steroidal anti-inflammatory drugs (NSAIDs) are extremely used in the treatment of pain and inflammation. These compounds non selectively inhibit the two isoforms of the cyclooxygenase (COX-1 and COX-2) and so prevent the metabolism of cellular arachidonic acid (AA) and the upregulation of prostaglandin formation, which in other respects lead to an increase of vascular permeability, edema, hyperalgesia, pyrexia and inflammation (1-5). 

Additionally, Leukotrienes, particularly LTB4 which are products of 5-LO together with decrease in prostaglandin formation are incriminated in the acute ulceration induced by NSAID’s. Accordingly, compounds that obtain dual inhibition of enzymes COX and 5-LO can show safer profile of activity and enhanced efficacy in the battle of pain in inflammatory diseases. 

**Figure 1:**
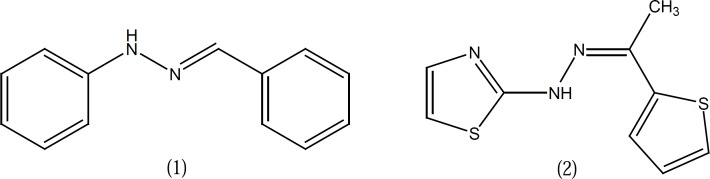
Structure of compounds 1, 2

Some reports suggest that the hydrazone moiety present in phenylhydrazone derivative 1 (Figure 1) is a pharmacophore group for the inhibition of COX and hydrazone type compounds such as compound 2 were depicted as dual COX/5-LO inhibitors (Figure 1) (3, 4). 

2-phenoxyphenyl ring system and it’s analogues has attracted some interests in medicinal chemistry and this scaffold have demonstrated a wide range of biological effects which include anticonvulsant, antimycobacterial, analgesic and anti-inflammatory activities (6- 11).

According to the above and on the basis of molecular hybridization approach which is based on the combination of different pharmacophores to produce a new hybrid molecule with improved activity, we aimed at the synthesis of new hybrid compounds containing both of hydrazone and 2-phenoxyphenyl structures in the hope of obtaining new potent analgesic agents with potential dual COX/5-LO inhibitory activity and lower side effects (12-15). 

## Experimental

*Chemistry*

**Figure 2:**
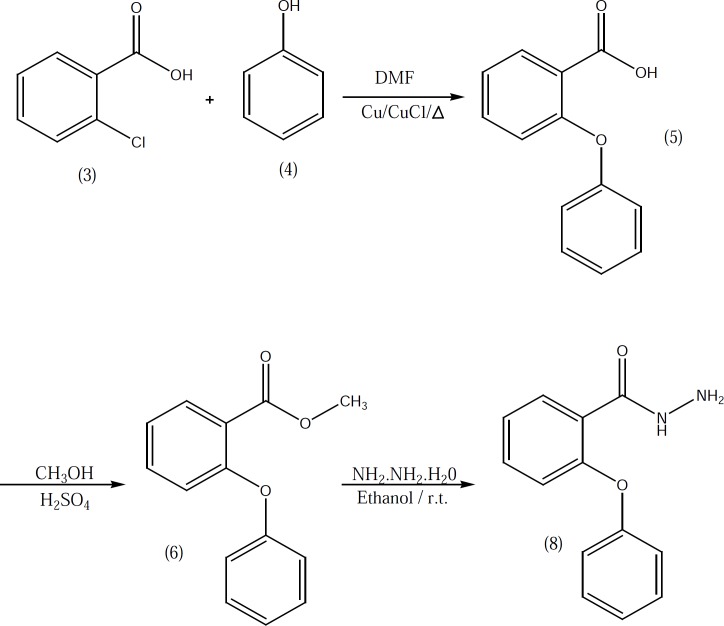
Synthesis of 2-phenoxybenzoic acid hydrazide (8

**Figure 3:**
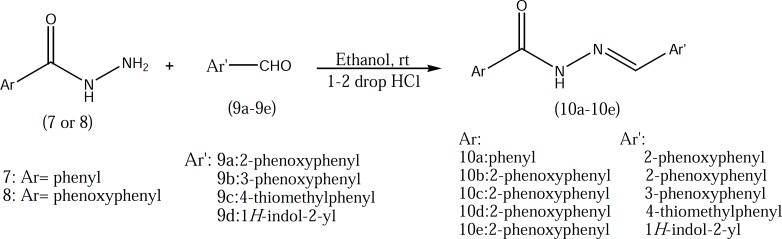
Synthesis of target compounds (hydrazide derivatives).

**Figure 4:**
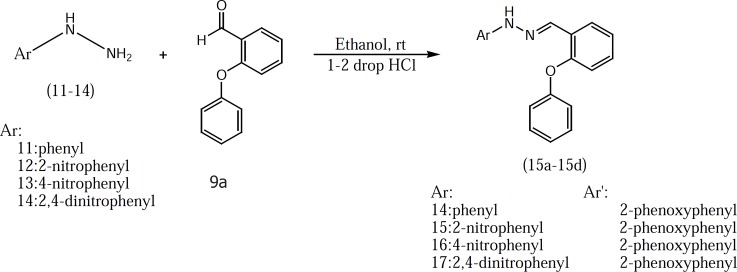
Synthesis of target compounds (hydrazine derivatives).

2-phenoxybenzoic acid hydrazide 8 which used in the preparation of compounds 10b-10e was synthesized according to our previously described method (Figure 2) (3). Target compounds were synthesized in one step shown in Figures 3 and 4. The designed compounds 10a-10e and 15a-15d were synthesized by acid-catalyzed condensation of hydrazides 7 and 8 or hydrazines 11-14 with corresponding aldehydes 9a-9d. The structures of the synthesized compounds were assigned on the basis of IR, 1H-NMR and Mass spectra. 

Chemicals were purchased from Merck Chemical company (Tehran, Iran) and ACROS company (Renningen, Germany). The melting points were determined in open capillary tubes and presented uncorrected. 1H-NMR spectra were obtained using a Bruker FT-400 spectrometer (Bruker, Rheinstetten, Germany). Tetramethylsilane was used as an internal standard. Mass Spectra were obtained using a Finnigan Mat TSQ-70 spectrometer at 70 eV (Finnigan Mat, Bremen, Germany). The IR spectra were obtained using a Nicolet FT-IR Magna 550 Spectrographs (KBr disks) (Nicolet, Madision, WI, USA). The target compounds were synthesized according to the Figures 3 and 4. 

**Table1:** Physical data of synthesized compounds

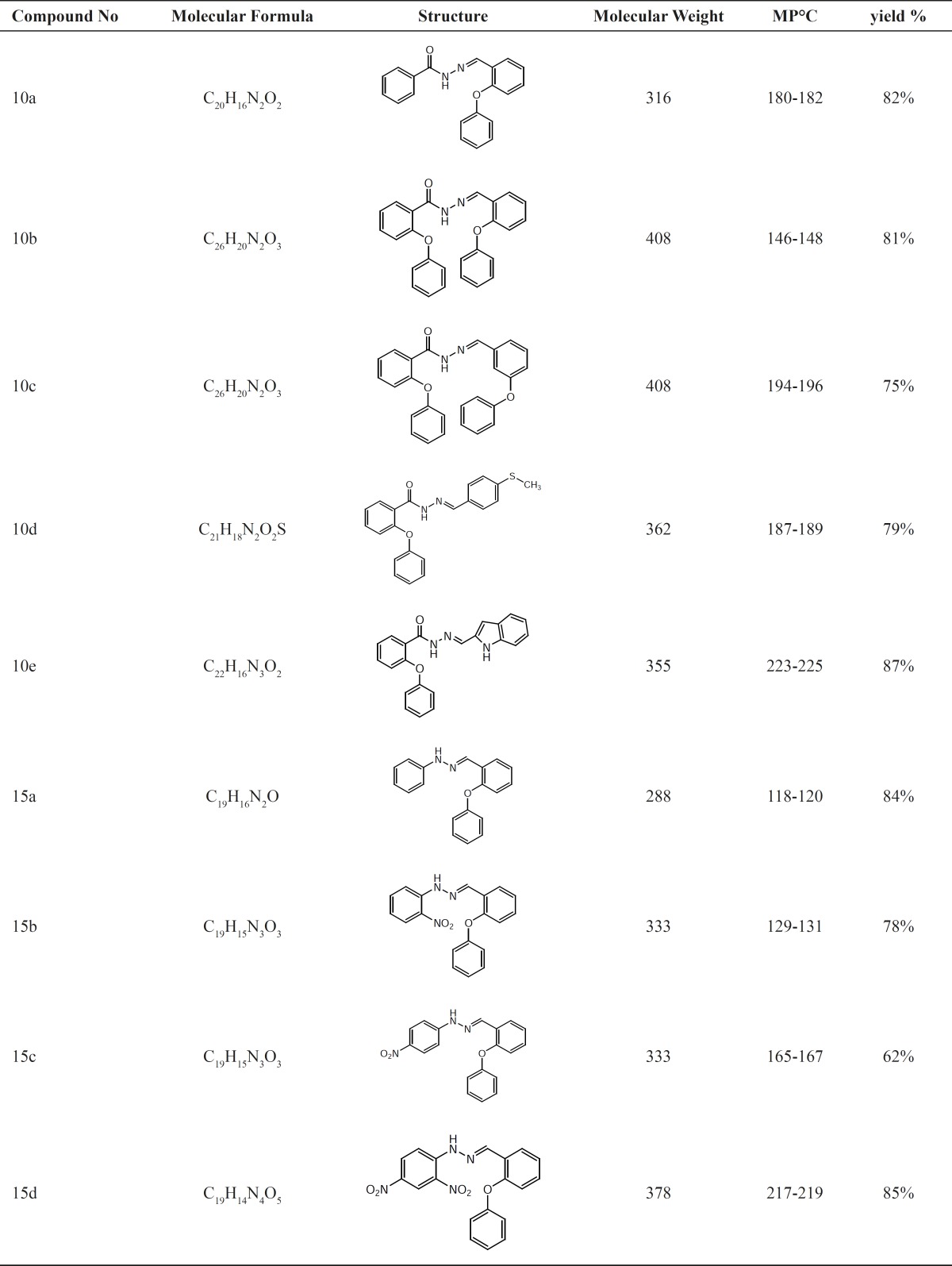

*General procedure for the synthesis of hydrazides (10b-10e) and hydrazines (15a-15d)*

A mixture of hydrazides 7-8 or hydrazines 11-14 (2mmol) and corresponding aldehydes 9a-9d (2.1mmol) in absolute ethanol (20ml) were stirred at room temperature for 1-5 hr in the presence of two drops of hydrochloric acid as a catalyst. The end of the reaction was observed by TLC, and then neutralized by 10% aqueous solution of sodium bicarbonate. The resulting precipitate was filtered, washed with 20 ml water and dried and crystallized by ethanol.

*Spectral data of selected compounds*

*N’-(2-phenoxybenzylidene)benzohydrazide (10a)*

1H-NMR (DMSO, d6) : 11.93 (s, 1H, NH), 8.78 (s, 1H, CH=N), 7.90 (d, J=6.8Hz, 2H, aromatic), 7.58-7.16 (m, 9H, aromatic), 7.01(d, J=7.6Hz, 2H, aromatic), 6.96(d, J=7.6Hz, 1H, aromatic). IR (KBr) : ν cm^-1^, 3310 (NH), 1682 (C=O), 1643(C=N). Mass (m/z): 316(M^+^ ,78), 121(100), 105 (64), 93 (55), 77 (95); Anal. Calcd. for C20H16N2O2: C, 75.93; H, 5.10; N, 8.86. Found: C, 75.64; H, 5.32; N, 8.72. 

*N ’ - ( 2 - p h e n o x y b e n z y l i d e n e ) - 2 - phenoxybenzohydrazide (10b)*

1H-NMR (DMSO, d6): 11.94 (s, 1H, NH), 8.58 (s, 1H, N=CH), 7.99(d, J=7.6Hz, 1H, aromatic), 7.85 (d, J=7.2Hz, 1H, aromatic), 7.66-6.84 (m, 16H, aromatic). IR (KBr): ν cm-1, 3290(NH), 1678(C=O), 1638(C=N). Mass (m/z): 408(M^+^ ,100), 197(74), 169(38), 93(61), 77(84); Anal. Calcd. for C26H20N2O3: C, 76.45; H, 4.94; N, 6.86. Found: C, 76.20; H, 5.22; N, 6.69.

*N’-(3-phenoxybenzylidene)-2-phenoxybenzohydrazide (10c)*

1H-NMR (DMSO, d6): 11.72 (s, 1H, NH), 8.27 (s, 1H, N=CH), 7.95(d, J=8.0Hz, 1H, aromatic), 7.69-6.94 (m, 17H, aromatic). IR (KBr): ν cm^-1^, 3320(NH), 1680 (C=O), 1640 (C=N). Mass (m/z): 408(M^+^ ,100), 213(81), 197(80), 169(51), 93 (78), 77(85); Anal. Calcd. for C_26_H_20_N_2_O_3_: C, 76.45; H, 4.94; N, 6.86. Found: C, 76.38; H, 5.17; N, 6.73.

*N’-(4-(methylthio)benzylidene)-2-phenoxybenzohydrazide (10d)*

1H-NMR (DMSO, d6): 11.81(s, 1H, NH), 7.93(d, J=7.8Hz, 1H, aromatic), 7.22, 7.60 (m, 7H, aromatic), 7.15-6.93(m, 5H, aromatic), 2.51(s, 3H, CH3). IR (KBr): ν cm^-1^, 3295(NH), 1685(C=O), 1646(C=N). Mass (m/z): 362(M^+^ ,100), 197(88), 120(49), 93(70), 77(65); Anal. Calcd. for C_21_H_18_N_2_O_2_S: C, 69.59; H, 5.01; N, 7.73. Found: C, 69.72; H, 5.24; N, 7.41.

*N’-((1H-indol-2-yl)methylene)-2-phenoxybenzohydrazide(10e)*

1H-NMR (DMSO , d6) : 13.46(s, 1H, NH), 12.1(s, 1H, NH), 8.21(s, 1H, N=CH), 7.90(d, J=7.6Hz, 1H, aromatic), 7.71-7.37(m, 9H, aromatic), 7.19-6.90(m, 4H, aromatic), IR (KBr) : ν cm^-1^, 3375, 3324(NH), 1685(C=O), 1645(C=N). Mass (m/z): 355(M^+^ , 58), 135(100), 120(87), 104(65), 77(82); Anal. Calcd. for C_22_H_17_N_3_O_2_: C, 74.35; H, 4.82; N, 11.82. Found: C, 74.01; H, 4.99; N, 11.51.

*2-(2-phenoxybenzylidene)-1-phenylhydrazine (15a)*

1H-NMR (DMSO, d6): 11.20(s, 1H, NH), 8.15(s, 1H, N=CH), 7.70-7.20(m, 11H, aromatic), 7.15-6.91(m, 3H, aromatic). IR (KBr): ν cm^-1^, 3335(NH), 1635(C=N). Mass (m/z): 288(M^+^ , 100), 93(79), 92(68), 77(95).; Anal. Calcd. for C_19_H_16_N_2_O: C, 79.14; H, 5.59; N, 9.72. Found: C, 79.43; H, 5.39; N, 9.88.

*2-(2-phenoxybenzylidene)-1-(2-nitrophenyl) hydrazine (15b)*

1H-NMR (DMSO, d6): 11.33(s, 1H, NH), 8.79(bs, 1H, aromatic), 8.10(s, 1H, CH=N), 7.97(d, J=8.0Hz, 1H, aromatic), 7.65-6.92(m, 11H, aromatic).

IR (KBr): ν cm^-1^, 3322(NH), 1630(C=N), 1359, 1548(NO2). Mass (m/z): 333(M^+^ , 100), 137(48), 93(80), 77(53); Anal. Calcd. for C_19_H_15_N_3_O_3_: C, 68.46; H, 4.54; N, 12.61. Found: C, 68.39; H, 4.31; N, 12.69.

*2-(2-phenoxybenzylidene)-1-(4-nitrophenyl) hydrazine (15c)*

1H-NMR (DMSO, d6): 11.42(s, 1H, NH), 8.71(d, J = 8.8Hz, 2H, aromatic), 8.15(s, 1H, CH = N), 7.79-7.18(m, 8H, aromatic), 7.11-6.95(m, 3H, aromatic). IR (KBr): ν cm^-1^, 3342(NH), 1639(C=N), 1352, 1543(NO_2_). Mass (m/z): 333(M^+^ , 100), 137(57), 93(74), 77(61); Anal. Calcd. for C_19_H_15_N_3_O_3_: C, 68.46; H, 4.54; N, 12.61. Found: C, 68.37; H, 4.542; N, 12.08.

*2-(2-phenoxybenzylidene)-1-(2,4-dinitrophenyl)hydrazine (15d)*

1H-NMR (DMSO , d6) : 11.54(s, 1H, NH), 9.10(s, 1H, aromatic), 8.78(d, J=8.0Hz, 1H, aromatic), 8.63(s, 1H, CH=N), 7.92(d, J=8.0Hz, 1H, aromatic), 7.77-7.20(m, 6H, aromatic), 7.15-6.93(m, 3H, aromatic). IR (KBr): ν cm^-1^, 3322(NH), 1634(C=N), 1359, 1548(NO_2_). Mass (m/z): 378(M^+^ , 100), 182(91), 167(50), 77(94); Anal. Calcd. for C_19_H_14_N_4_O_5_: C, 60.32; H, 3.73; N, 14.81. Found: C, 60.16; H, 3.38; N, 14.56.

*Pharmacological evaluation*

Male NMRI mice weighting 20-25 g (from animals house of Faculty of Pharmacy, TUMS) were used for abdominal constriction test (writhing test). The animal were housed in colony cages and conditions of constant temperature (22 ± 2°C) and a 12 h hlight/dark schedule and allowed free access to standard diet and tap water except during the experiment. The animals were allowed to habituate to the laboratory environment for 2h before the experiments were initiated. All ethical manners for use of laboratory animals were considered carefully and the protocol of study was approved by TUMS ethical committee. The compounds were administered intraperitoneally (IP) (30 mg/kg; 0.2 ml/20g) as a suspension in saline and tween 80 (4%w/ν). Mefenamic acid (Hakim Pharmaceutical Co) (30 mg/kg, IP) was used as standard drug under the same conditions. The control group received vehicle (0.2 ml/20g, IP) alone 

*Analgesic activity*

The analgesic activity was determined in vivo by the abdominal constriction test induced by acetic acid(0.6%; 0.1 mL/10 g) in mice (4). An acetic acid solution was administered IP 30 minutes after administration of compounds. Antinociception was recorded by counting the number of writhings immediately after injection of acetic acid during 30 minutes. The analgesic activity was expressed as the percentage of inhibition of constrictions when compared with the vehicle control group Table 2 (16). Percentage of analgesic activity (PA) was calculated according to the formula: PA= (1-T/C)× 100 where C and T are number of writhes in control and drug treated group, respectively.

**Table 2:** Effects of Compounds 10a-10e and 15a-15d and mefenamic acid in the abdominal constrictions induced by acetic acid in mice

**Compound**	**Dose** **(mg/kg)**1	**Constriction No.** **(mean ± SEM)**	**Inhibition** **(%)**2	**p-value**
Control	-	63.5 ± 16.77	-	-
mefenamic acid	30	8.17 ± 1.35	87.13	p < 0.001
10a	30	49.50 ± 9.731	22.04	p > 0.05
10b	30	12.33 ± 2.58	80.58	p < 0.001
10c	30	22.33 ± 3.02	54.85	p < 0.001
10d	30	50.67 ± 5.48	4.4	p > 0.05
10e	30	43.33 ± 1.78	26.25	p < 0.01
15a	30	38.54 ± 1.18	32.8	p < 0.001
15b	30	3.83 ± 0.91	91.33	p < 0.001
15c	30	19.17 ± 0.79	67.71	p < 0.001
15d	30	43.67 ± 1.11	31.22	p < 0.01

1 number of animals in each group n= 6; 2 % inhibition obtained by comparison with vehicle control group.

*Statistical analysis*

Ststistical analysis was done by one-way analysis of variance (ANOVA) and followed by Tukey multiple comparison test. Differences with p < 0.05 between experimental groups were considered statistically significant.

## Results and Discussion

In this study, a new series of hydrazide and hydrazine derivatives were synthesized and evaluated for analgesic activity using the acetic acid induced mice abdominal constriction test and the results are shown in Table 2. Except compounds 10a and 10d all of them induced significant reduction in the writhing response in comparison to control and among them compound 15b showed higher inhibitory effect in comparison to mefenamic acid. 

As shown in Table 2, the best derivatives were 10b, 10c, 15b and 15c which belong to both hydrazide and hydrazine compounds.

So withdrawing of carbonyl moiety doesn’t have a deleterious effect on the analgesic potency. It has been observed that attachment of phenoxy group in both 2 and 3 positions of phenyl ring can result good compounds. Since compounds 10a, 10d and 10e showed weak activity, we can conclude that in hydrazide derivatives existence of phenoxy moiety in both side of the molecule is essential. Attachment of electronwithrawing groups like Nitro substitution on ortho or para position of phenyl ring in hydrazines can improve the activity but substitution of both positions by these groups has a decrescent effect on analgesic activity. In conclusion several synthesized compounds showed comparable or better analgesic activity than the reference drug.

## Conclusion

New hydrazide and hydrazone derivatives were synthesized and their structures were confirmed by spectral data. Their analgesic activity was tested by writhing test and most of them were active analgesic agents. Since in vivo activity depend on highly complex physiological interactions, therefore the SAR’s which were presented earlier are just probable and their validity can’t be claimed absolutely.
